# Insecticide resistance of *Anopheles sinensis* after elimination of malaria in Henan Province, China

**DOI:** 10.1186/s13071-023-05796-z

**Published:** 2023-06-02

**Authors:** Zhi-quan He, Ya-bo Hu, Dan Wang, Yu-ting Liu, Cheng-yun Yang, Dan Qian, Rui-min Zhou, De-ling Lu, Su-hua Li, Ying Liu, Hong-wei Zhang

**Affiliations:** 1 Department of Parasite Disease Control and Prevention, Henan Center for Disease Control and Prevention, Zhengzhou, China; 2 Henan Provincial Medical Key Laboratory of Parasitic Pathogen and Vector, No. 105 South Agricultural Road, Zhengdong New District, Zhengzhou, 450016 China; 3 Zhengzhou Second Hospital, No. 90 Hanghai Middle Road, Erqi District, Zhengzhou, 450016 China; 4 College of Public Health, Zhengzhou University, No.100 Science Avenue, High-Tech District, Zhengzhou, 450016 China

**Keywords:** *Anopheles sinensis*, Knockdown resistance, Acetylcholinesterase-1, Population genetic characteristics, Henan Province

## Abstract

**Background:**

Historically, malaria due to *Plasmodium vivax* has been epidemic in Henan Province, China, with *Anopheles sinensis* as the main vector. The most effective measures to prevent malaria transmission are based on vector control through the use of insecticides. However, insecticides exert a strong selective pressure on mosquito populations for insecticide resistance. The aim of this study was to investigate the susceptibility profile and population genetic characteristics of *An. sinensis* to provide basic data and scientific guidance for the study of resistance mechanisms and the control of *An. sinensis* in Henan Province.

**Methods:**

Adult *Anopheles* mosquitoes were collected at sites near local farmers' sheepfolds, pigsties and/or cowsheds located in Pingqiao, Xiangfu, Xiangcheng and Tanghe counties/districts of Henan Province during July–September 2021 for insecticide susceptibility testing. Molecular identification of collected mosquitoes as belonging to genus *Anopheles* was by PCR, and the frequencies of mutations in the knockdown resistance gene (*kdr*) and acetylcholinesterase-1 gene (*ace-1*) were detected using gene amplification. The mitochondrial DNA cytochrome oxidase subunit I (COI) gene was amplified in deltamethrin-resistant and deltamethrin-sensitive mosquitoes to analyze the genetic evolutionary relationship.

**Results:**

A total of 1409 *Anopheles* mosquitoes were identified by molecular identification, of which 1334 (94.68%) were *An. sinensis*, 28 (1.99%) were *An. yatsushiroensis*, 43 (3.05%) were *An. anthropophagus* and four (0.28%) were *An. belenrae*. The 24-h mortality rates of *An. sinensis* in Pingqiao, Tanghe, Xiangcheng and Xiangfu counties/districts exposed to deltamethrin were 85.85%, 25.38%, 29.73% and 7.66%, respectively; to beta-cyfluthrin, 36.24%, 70.91%, 34.33% and 3.28%, respectively; to propoxur, 68.39%, 80.60%, 37.62% and 9.29%, respectively; and to malathion, 97.43%, 97.67%, 99.21% and 64.23%, respectively. One mutation, G119S, was detected in the *ace-1* gene. The frequencies of the main genotypes were 84.21% of specimens collected in Xiangfu (G/S), 90.63% of speciments collected in Xiangcheng (G/G) and 2.44% of speciments collected in Tanghe (S/S). Significantly higher G119S allele frequencies were observed in both propoxur- and malathion-resistant mosquitoes than in their sensitive counterparts in the Tanghe population (*P* < 0.05). Three mutations, L1014F (41.38%), L1014C (9.15%) and L1014W (0.12%), were detected in the *kdr* gene. The genotypes with the highest frequency in the populations of *An. sinensis* in Xiangfu and Tanghe were the mutant TTT (F/F) and wild-type TTG (L/L), at 67.86% (57/84) and 74.29% (52/70), respectively. In Pingqiao and Xiangfu, higher frequencies of the L1014F allele and lower frequencies of the L1014C allele were observed in mosquitoes resistant for beta-cyfluthrin than in those which were sensitive for this insecticide (*P* < 0.05). The results of Tajima's *D* and of Fu and Li's *D* and *F* were not significantly negative (*P* > 0.10), and each haplotype was interlaced and did not form two distinct branches.

**Conclusions:**

High resistance to pyrethroids and propoxur was observed at four sites, but the resistance to malathion varied according to the location. *Anopheles belenrae* and the L1014W (TGG) mutation in *An. sinensis* were first discovered in Henan Province. The deltamethrin-resistant and deltamethrin-sensitive mosquito populations showed no genetic differentiation. The generation of resistance might be the result of the combination of multiple factors.

**Graphical Abstract:**

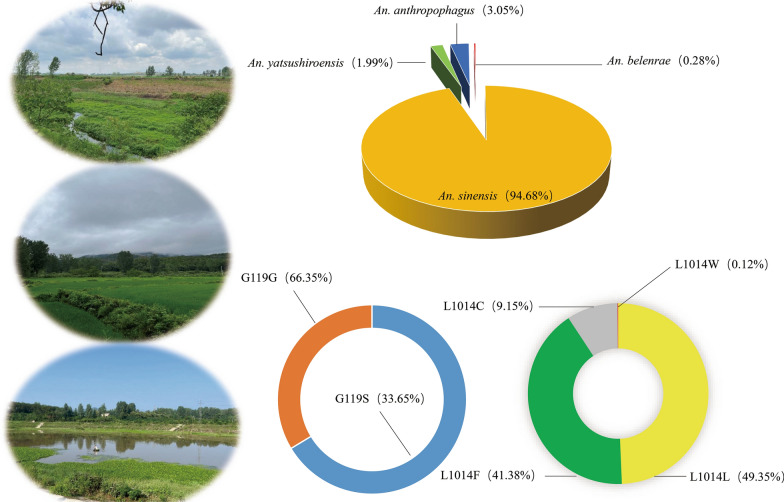

**Supplementary Information:**

The online version contains supplementary material available at 10.1186/s13071-023-05796-z.

## Background

Malaria, caused by infection with *Plasmodium* spp., is one of the most significant life-threatening infectious diseases in humans worldwide. According to the WHO Malaria Report 2022, the total number of deaths due to malaria worldwide reached 619,000 in 2021, with approximately 247 million reported malaria cases [1]. *Anopheles sinensis* is the predominant* Anopheles* species in Henan Province, China and the main vector of the malaria parasite* Plasmodium vivax* [2]. In 2006, there was an outbreak of malaria caused by *P. vivax* in Yongcheng county, Henan Province [3]. Malaria vectors are known to still exist in the original malaria-endemic areas and if there are also imported malaria cases, the risk of a malaria outbreak is extremely high. It has become an important measure to control the population density of *An. sinensis* and other malaria vectors and prevent secondary transmission caused by imported cases [4, 5]. Indigenous malaria transmission has been effectively controlled, and China officially obtained the WHO certification for malaria elimination in 2021. With the development of global trade and the transnational economy, infections imported from overseas have become the main source of malaria cases in Henan Province. Imported malaria has introduced new challenges to achieving the overall elimination of malaria [6–8].

Insecticides have been the most important measure to control the number of mosquitoes. Because of their broad-spectrum, efficiency and durable characteristics, insecticides are widely used across the globe [9]. Five major categories of insecticides have been applied: pyrethroids, organochlorines, carbamates, neonicotinoids and organophosphates [10]. Pyrethroids are emerging as the predominant insecticides for vector control because of their short residual action, high efficacy against mosquito vectors and low toxicity to humans [11]. The main targets of insecticides on mosquito vectors are the sodium ion channels (SC), acetylcholinesterase (AchE) and the gamma-aminobutyric (GABA) receptor-chloride channel complex [12]. Among these, mutations in the genes associated with the sodium channel in the axon produce knockdown resistance (*kdr*). The mutation at codon 1014 of the knockdown gene (*kdr*) in the IIS6 region of the sodium channel is also known as the *kdr* mutation [13, 14]. Four types of *kdr* mutations have been identified: (i) the L1014C (TTG → TGT) mutation leads to a change from leucine to cysteine substitution; (ii) the L1014W (TTG → TGG) mutation leads to a leucine to tryptophan substitution; and (iii) two L1014F (TTG → TTT, and TTG → TTC) mutations lead to a leucine to phenylalanine substitution. A mutation at codon 119 of the acetylcholinesterase-1 gene (*ace-1*) leads to a single amino acid substitution of glycine to serine in the binding pocket of acetylcholinesterase, conferring resistance to organophosphates and carbamates [15].

 Understanding the genetic differentiation of the population helps to infer the current situation and possible diffusion path of insecticide resistance, thus guiding the use of insecticides [16]. Population genetics, based on the characteristics of population genetic structure, analyzes the population evolutionary process, studies the genetic structure and change dynamics of organisms and explains the differences between populations. As an important component of the mitochondrial gene, the cytochrome oxidase subunit I (COI) gene has rich variation and is often used for phylogenetic and population genetic diversity analysis. The mitochondrial DNA (mtDNA)-COI gene sequence has been used in population genetic analyses due to its simple structure, fast evolutionary rate and extremely low probability of recombination [17–19].

Although the insecticide resistance of *An. sinensis* in Henan Province has been studied, little resistance monitoring data from recent years are available [20–22]. Therefore, using previous monitoring data, we have studied the insecticide resistance of *An. sinensis* in four regions of Henan Province, with the aim to determine the insecticide resistance status of *An. sinensis* in Henan Province. To further understand the current situation of target resistance and population genetic characteristics of *An. sinensis* in Henan Province, we selected samples of *An. sinensis* to detect the mutation and frequency of the *kdr* and *ace-1* genes, and carried out population genetic analysis of sensitive and resistant mosquitoes based on the mtDNA-COI gene, providing a scientific basis for guiding the control measures required for *An. sinensis* and basic data for further study of the population genetic characteristics of *An. sinensis* in China.

## Methods

### Study sites and mosquito collection

This study on the insecticide resistance of mosquitoes was carried out during July–September 2021 in four districts/counties of Henan Province, namely Pingqiao, Xiangfu, Xiangcheng and Tanghe. Adult *Anopheles* mosquitoes were collected using a Maxttrac Mosquito Trap (Shanghai Daolan Industrial Co., Ltd, China) near local farmers' sheepfolds, pigsties and/or cowsheds. Collected mosquitoes were transported to the insectary of the Henan Provincial Center for Disease Control and Prevention (CDC) where they were acclimatized under standard conditions (27 ± 1 °C, and 80% relative humidity [RH]) and provided with 8% glucose solution until the following day when they were used in the experiments. All collected mosquitoes were morphologically identified to their respective complexes and groups [23].

### Insecticide susceptibility test

In accordance with the standard WHO insecticide susceptibility tube-test procedures [24], adult female mosquitoes were used to conduct bioassays to determine susceptibility to four insecticides (supplied by China CDC): malathion (5%), deltamethrin (0.05%), beta-cyfluthrin (0.15%) and propoxur (0.1%). As controls, papers treated with olive oil, silicone oil, silicone oil and olive oil, respectively, were used. For each insecticide, 120 female mosquitoes were tested, with 20 mosquitoes per tube and two control tubes without insecticide. The number of knockdown mosquitoes was recorded during the exposure period at 10, 15, 20, 30, 40, 50 and 60 min. After 60 min of exposure, all mosquitoes were transferred to holding tubes, provided with 8% sugar water and placed under conditions of 26 °C and 80% RH. At the end of this 24-h recovery period, the number of dead mosquitoes was counted and recorded. After the bioassays, all mosquitoes were preserved individually in 1.5-ml Eppendorf tubes for further molecular analyses.

### DNA extraction and molecular identification

The mosquitoes collected in the field and morphologically identified as *An. sinensis* were tested for insecticide resistance and then their identity molecularly confirmed. One leg of each mosquito was used for DNA extraction with the Animal Tissue Genomic DNA Extraction Kit (Xi’an Tianlong Science & Technology Co., Ltd, Xi’an City, China). More precisely, one leg was placed in 100-μl aliquot of tissue lysis solution and fully ground using an electric tissue grinder. Then, 200 μl of tissue lysis solution and 40 µl of protein K solution were added and mixed by vortexing. An automatic nucleic acid extractor (Qiagen, Hilden, Germany) was used for automatic DNA extraction. Molecular identification of *An. sinensis* was performed by using species-specific primers and amplification of internal transcribed spacer 2 (ITS2) [25]. The samples identified as *An. sinensis* were again used for subsequent gene amplification and data analysis.

### Detection of *ace-1 and kdr* mutations

To determine point mutations of the *ace-1* gene at codon 119, a 193-bp fragment was amplified in *An. sinensis* using primers *ace-1*-F (467F; 5’-GTGCGACCATGTGGAACC-3’) and *ace-1*-R (660R; 5’-ACCACGATCACGTTCTCCTC-3’) [26]. Amplification reactions were performed in a 25-µl reaction volume containing 2 µl of the DNA sample, 12 µl of a 2 × Go Taq Green Master Mix (Promega Inc., Madison, WI, USA), 2 µl of target primers and 9 µl of ddH_2_O. PCR cycling consisted of 1 cycle at 95 °C for 3 min, followed by 35 cycles of 94 °C for 30 s, 55 °C for 30 s and 72 °C for 45 s, with a final extension at 72 °C for 6 min. Sequencing was performed by Shanghai DNA Bio Technologies Co., Ltd. (Shanghai, China). Genotyping of point mutations of the *kdr* gene at codon 1014 was conducted as previously described [11].

### COI gene amplification of deltamethrin-resistant and -sensitive mosquitoes

To clarify the genetic evolutionary relationship between resistant and sensitive mosquitoes of *An. sinensis*, the mtDNA-COI gene was amplified in deltamethrin-resistant and deltamethrin-sensitive *An. sinensis* using the forward primer GGTCAACAAATCATAAAGATATTGG and the reverse primer TAAACTTCAGGGTGACCAAAAAATCA, following the methods previously described by Liang et al. [27]. All PCR products were analyzed by 2% agarose gel electrophoresis and the purified products were sequenced using an ABI 3730XL automated sequencer (Applied Biosystems®, Thermo Fisher Scientific, Waltham, MA, USA.

### Statistical analysis

The mortality rates of the test and control samples were calculated after the bioassay for each insecticide group. If the control mortality was > 20%, the tests were discarded. When control mortality was 5% and 20%, then the observed mortality was corrected using Abbott’s formula [28]. Mosquito resistance status was interpreted in accordance with WHO guidelines as: (i) confirmed resistance (CR), mortality rate < 90%; (ii) possible resistance (PR), mortality rate between 90% and 97%; and (iii) susceptibility (S), mortality rate ≥ 98% [24]. Statistical analyses were performed using SPSS version 21.0 software (IBM Corp., Armonk, NY, USA). The mortality rates of *An. sinensis* at the survey sites, *ace-1* and *kdr* mutation rates and gene mutation rates of the different populations were calculated using the Chi-square (*χ*^2^) test. Nucleotide polymorphism, natural selection and population differentiation were described according to He et al. [29]. In addition, we constructed a haplotype network diagram among the different geographical populations based on the median connection method, with the aim to analyze the genetic relationship among different geographical populations using PopArt software. A *P* value of < 0.05 was considered to indicate significance.

## Results

### Molecular identification of *Anopheles* species and insecticide susceptibility test

The molecular identification of all samples used for insecticide resistance determination was carried out based on the length of the electrophoresis fragments of the PCR product. A total of 1409* Anopheles* mosquitoes were identified by species-specific PCR, of which 1334 (94.68%) were *An. sinensis*, 28 (1.99%) were *An. yatsushiroensis*, 43 (3.05%) were *An. anthropophagus* and four (0.28%) were *An. belenrae* (Table 1). Analysis of the insecticide resistance of the 1334 *An. sinensis* mosquitoes showed that the 24-h mortality rates in mosquitoes exposed to deltamethrin, beta-cyfluthrin and propoxur ranged from 3.28% to approximately 85.85%, which reached the CR level. The 24-h mortality rate of *An. sinensis* to malathion in Pingqiao and Tanghe was 97.43% and 97.67%, respectively, which reached the PR level. The 24-h mortality rates for malathion in Xiangfu and Xiangcheng were 64.23% (CR) and 99.21% (S), respectively. The difference between the knockdown rate and 24-h mortality of the different insecticides at the four sampling locations was statistically significant (χ^2^ = 32.635 to approx. 143.433; *P* < 0.05). For beta-cyfluthrin, deltamethrin, propoxur and malathion, the median knockdown time for 50% of the tested mosquitoes (KT_50_) ranged from 29.81 min to approximately 315.73 min in Pingqiao, Tanghe, Xiangcheng and Xiangfu. For beta-cyfluthrin and deltamethrin, the KT_50_ could not be calculated due to the low knockdown rate in Xiangfu (Table 2).

**Table 1:** Sampling locations and molecular identification of *Anopheles* species collected in Henan Province, China

Sampling sites/populations	Longitude and latitude	Collection date	Sites	*Anopheles sinensis*, *n* (%)	*Anopheles belenrae*, *n* (%)	*Anopheles yatsushiroensis*, *n* (%)	*Anopheles anthropophagus*, *n* (%)	Sample size (*n*)
Pingqiao	114.12°E, 32.10°N	July 2019	Pigsties	349 (99.15)	2 (0.57)	1 (0.28)	0 (0.00)	352
Xiangfu	114.44°E, 34.76°N	August 2019	Pigsties and sheepfolds	353 (100)	0 (0.00)	0 (0.00)	0 (0.00)	353
Xiangcheng	113.48°E, 33.85°N	September 2019	Sheepfolds	368 (100)	0 (0.00)	0 (0.00)	0 (0.00)	368
Tanghe	112.83°E, 32.70°N	September 2019	Sheepfolds and Cowsheds	264 (78.57)	2 (0.60)	27 (8.04)	43 (12.80)	336
Total	–	–	–	1334 (94.68)	4 (0.28)	28 (1.99)	43 (3.05)	1409

**Table 2:** Adjusted mortality rates and knockdown times of *Anopheles sinensis* exposed to four insecticides in Henan Province

Sampling sites/populations	Insecticide	Sample size (*n*)	Knockdown in 1 h (*n*)	Rate of knockdown in 1 h (%)	KT_50_(95% CI), per min	Deaths in 24 h (*n*)	Adjusted rate of deaths in 24 h (%)	Status^a^
Pingqiao	Beta-cyfluthrin	86	21	24.42	315.73 (143.53,2848.63)	37	36.24	CR
	Deltamethrin	87	66	75.86	33.35 (30.21,37.10)	76	85.85	CR
	Propoxur	87	42	48.28	70.77 (56.28,102.16)	63	68.39	CR
	Malathion	89	59	66.29	29.81 (25.28, 35.59)	87	97.43	PR
	Control	55	0	–	–	7	12.73^c^	–
Tanghe	Beta-cyfluthrin	55	25	45.45	68.18 (55.03, 96.65)	39	70.91	CR
	Deltamethrin	113	23	20.35	101.10 (81.66,148.67)	39	25.38	CR
	Propoxur	47	34	72.34	36.60 (33.37,40.33)	39	80.60	CR
	Malathion	49	45	91.84	32.09 (29.73,34.53)	48	97.67	PR
	Control	49	0	–	–	6	12.24^c^	–
Xiangcheng	Beta-cyfluthrin	67	25	37.31	96.69 (69.21,183.34)	23	34.33^d^	CR
	Deltamethrin	74	8	10.81	237.80 (125.09,1719.26)	22	29.73^d^	CR
	Propoxur	101	51	50.50	55.64 (49.13,65.57)	38	37.62^d^	CR
	Malathion	126	104	82.54	32.33 (27.80,37.43)	125	99.21^d^	S
	Control	60	0	–	–	2	3.33^c^	–
Xiangfu	Beta-cyfluthrin	81	2	2.47	NC^b^	8	3.28	CR
	Deltamethrin	86	2	2.33	NC	12	7.66	CR
	Propoxur	84	19	22.62	159.36 (101.27,412.00)	13	9.29	CR
	Malathion	102	16	15.69	133.81 (96.65,254.71)	68	64.23	CR
	Control	44	0	–	–	3	6.82^c^	–

*CI* Confidence interval, * KT*_*50*_ median knockdown time for 50% of the tested mosquitoes, *NC* not calculated,

^a^CR, Confirmed resistance (mortality < 90%); PR, possible resistance (90% ≤ mortality ≤ 97%); S, susceptible (mortality ≥ 98%)

^b^Unable to calculate mean KT_50_ due to low knockdown rate

^c^Mean mortality rates in the controls

^d^Mean unadjusted rate of death in 24 h

### Association between *ace-1* genotype and insecticide resistance

Mosquitoes molecularly identified as *An. sinensis* were used for amplification of *ace-1*. A total of 315 mosquitoes, including propoxur- and malathion-resistant and -sensitive mosquitoes, were used in this study. After splicing the sequencing results, a 193-bp-long gene sequence was obtained. Three genotypes of the *ace-1* gene in different populations of *An. sinensis* were detected through sequence alignment: GGC (G/G), AGC (S/S) and GGC/AGC (G/S). Among these, G/G is the homozygous wild type, S/S is a homozygous mutant and G/S is a heterozygous mutant. Three genotypes were detected in three populations: Pingqiao, Tanghe and Xiangfu. Only two genotypes were detected in Xiangcheng, with no mosquito with genotype S/S found at this location. The main genotype of the Pingqiao and Xiangfu populations was G/S, with frequencies of 68.67% (57/83) and 84.21% (80/95), respectively. The Tanghe and Xiangcheng populations were genotype-based, with G/G having the highest frequency in both populations at 73.17% (30/41) and 90.63% (87/96), respectively, and genotype S/S only found in the Tanghe population, with a frequency of only 2.44% (1/41). Two alleles were found in the four sampling populations of *An. sinensis* in Henan Province: wild-type G119G and mutant-type G119S, with a total mutation rate of 33.65%. Significantly higher G119S allele frequencies were observed in both propoxur- and malathion-resistant mosquitoes than in propoxur- and malathion-sensitive mosquitoes in the Tanghe population (*P* < 0.05), but this distribution was not observed in the other three populations. Table 3 presents detailed data on the relationship between the *ace-1* mutant gene and insecticide resistance in* An. sinensis.*

**Table 3:** Relationship between the *ace-1* mutant gene and insecticide resistance in* An. sinensis*

Sampling sites/populations	Insecticides	Status^a^	Sample size (*n*)	*ace-1* genotype (*n*)^b^			Allelic gene frequency		Odds ratio (95% *CI*) G119S
				G/G	S/S	G/S	G119G, *n* (%)	G119S, *n* (%)	
Pingqiao	Propoxur	CR	18	4	4	10	18 (50.00)	18 (50.00)	0.52 (0.23, 1.20)
		S	32	0	10	22	22 (34.38)	42 (65.63)	
	Malathion	CR	2	0	1	1	1 (25.00)	3 (75.00)	4.75(0.47, 48.34)
		S	31	7	0	24	38 (61.29)	24 (38.71)	
	Total	–	83	11	15	57	79 (47.59)	87 (52.41)	–
Tanghe	Propoxur	CR	7	0	1	6	6 (42.86)	8 (57.14)	17.33* (2.91, 103.38)
		S	14	12	0	2	26 (92.86)	2 (7.14)	
	Malathion	CR	1	0	0	1	1 (50.00)	1 (50.00)	37.00* (1.22, 1119.83)
		S	19	18	0	1	37 (97.37)	1 (2.63)	
	Total	–	41	30	1	10	70 (85.37)	12 (14.63)	–
Xiangcheng	Propoxur	CR	33	29	0	4	62 (93.94)	4 (6.06)	3.81(0.41, 35.05)
		S	30	29	0	1	59 (98.33)	1 (1.67)	
	Malathion	CR	1	0	0	1	1 (50.00)	1 (50.00)	20.33(1.00, 410.20)
		S	32	29	0	3	61 (95.31)	3 (4.69)	
	Total	–	96	87	0	9	183 (95.31)	9 (4.69)	–
Xiangfu	Propoxur	CR	32	0	4	28	28 (43.75)	36 (56.25)	1.50(0.60, 3.75)
		S	13	1	0	12	14 (53.85)	12 (46.15)	
	Malathion	CR	18	0	5	13	13 (36.11)	23 (63.89)	1.66 (0.72, 3.84)
		S	32	2	3	27	31 (48.44)	33 (51.56)	
	Total	–	95	3	12	80	86 (45.26)	104 (54.74)	–
Total	–	–	315	131	28	156	418 (66.35)	212 (33.65)	–

*ace-1* Acetylcholinesterase-1 gene

*P < 0.05

^a^CR, Confirmed resistance (mortality < 90%); PR, possible resistance (90% ≤ mortality ≤ 97%); S, susceptible (mortality ≥ 98%)

^b^G/G refers to the wild-type *ace-1* genotype (GGC), S/S refers to the homozygous *ace-1* mutation (AGC), G/S is heterozygote for *ace-1*(GGC/AGC)

### Association between *kdr* genotype and insecticide resistance

A 325-bp fragment that included position 1014 was successfully sequenced from 339 deltamethrin and beta-cyfluthrin-resistant and deltamethrin and beta-cyfluthrin-sensitive *An. sinensis*. The mutation frequency at codon 1014 of the *kdr* gene at the four collection sites was 68.83% (Pingqiao), 18.04% (Tanghe), 17.70% (Xiangcheng) and 98.05% (Xiangfu). Ten genotypes of the *kdr* gene in the different populations of *An. sinensis* were detected through sequence alignment: TTG (L/L), TTT (F/F), TGT (C/C), TTG/TGT (L/C), TGG/TTT (W/F), TTT/TGT (F/C), TGT/TTC (C/F), TTG/TTT (L/F), TTG/TTC (L/F) and TTT/TTC (F/F). Among these, TTG is the wild type; the other nine are mutations, with TTT and TGT being homozygous mutations, and the remaining seven genotypes being heterozygous mutations. The highest number of genotypes was detected in the Pingqiao sample, including nine genotypes. The genotypes with the highest frequency in the Pingqiao and Xiangfu populations of *An. sinensis* were the mutant TTT (F/F) genotype, at 32.18% (28/87) and 67.86% (57/84), respectively. The highest frequency of the wild-type TTG (L/L) genotype was found in the Tanghe and Xiangcheng populations, at 74.29% (52/70) and 67.35% (66/98), respectively (Additional file 1: Table S1). As shown in Table 4, the total mutation rate of the *kdr* gene was 50.65%, with the L1014F mutation having the highest frequency (41.38%); the frequency of the L1014C mutation was 9.15%, and the L1014W mutation had the lowest frequency (0.12%). A significantly higher frequency of the L1014F allele was observed in beta-cyfluthrin-resistant mosquitoes than in their sensitive counterparts in the Pingqiao, Tanghe and Xiangfu populations, but a significantly lower frequency of the L1014C allele was observed in beta-cyfluthrin-resistant mosquitoes than in their sensitive counterparts in the Pingqiao and Xiangfu populations (*P* < 0.05).

**Table 4:** Relationship between the *kdr* mutant gene and insecticide resistance in* An. sinensis*

Sampling sites/populations	Insecticide	Status^a^	Sample size (*n*)	Allele frequency, *n* (%)^b^				Odds ratio (95% *CI*)		
				L1014L	L1014F	L1014C	L1014W	L1014F	L1014C	L1014W
Pingqiao	Beta-cyfluthrin	CR	34	18 (26.47)	47 (69.12)	3 (4.41)	0 (0.00)	2.80* (1.33,5.88)	0.20* (0.05,0.78)	NC
		S	27	19 (35.19)	24 (44.44)	10 (18.52)	1 (1.85)			
	Deltamethrin	CR	11	8 (36.36)	11 (50.00)	3 (13.64)	0 (0.00)	1.00 (0.33,3.00)	0.52 (0.12,2.29)	NC
		S	15	8 (26.67)	15 (50.00)	7 (23.33)	0 (0.00)			
	Total	–	87	53 (31.17)	97 (53.39)	23 (14.97)	1 (0.46)	–	–	–
Tanghe	Beta-cyfluthrin	CR	15	19 (63.33)	9 (30.00)	2 (6.67)	0 (0.00)	5.86* (1.43,23.96)	3.07 (0.27,35.49)	NC
		S	22	40 (90.91)	3 (6.82)	1 (2.27)	0 (0.00)			
	Deltamethrin	CR	24	38 (79.17)	7 (14.58)	3 (6.25)	0 (0.00)	NC	1.13 (0.11,11.66)	NC
		S	9	17 (94.44)	0 (0.00)	1 (5.56)	0 (0.00)			
	Total	–	70	114 (81.96)	19 (12.85)	7 (5.19)	0 (0.00)	–	–	–
Xiangcheng	Beta-cyfluthrin	CR	24	39 (81.25)	6 (12.50)	3 (6.25)	0 (0.00)	1.43 (0.38,5.44)	1.40 (0.22,8.80)	NC
		S	22	38 (86.36)	4 (9.09)	2 (4.55)	0 (0.00)			
	Deltamethrin	CR	31	50 (80.65)	8 (12.90)	4 (6.45)	0 (0.00)	1.41 (0.40,5.01)	0.66 (0.15,2.78)	NC
		S	21	34 (80.95)	4 (9.52)	4 (9.52)	0 (0.00)			
	Total	–	98	161 (82.30)	22 (11.00)	13 (6.69)	0 (0.00)	–	–	–
Xiangfu	Beta-cyfluthrin	CR	32	3 (4.69)	58 (90.63)	3 (4.69)	0 (0.00)	4.39* (1.14,16.96)	0.11* (0.02,0.52)	NC
		S	8	0 (0.00)	11 (68.75)	5 (31.25)	0 (0.00)			
	Deltamethrin	CR	32	2 (3.13)	60 (93.75)	2 (3.13)	0 (0.00)	NC	NC	NC
		S	12	0 (0.00)	24 (100.00)	0 (0.00)	0 (0.00)			
	Total	–	84	5 (1.95)	153 (88.28)	10 (9.77)	0 (0.00)	–	–	–
Total	–	–	339	333 (49.35)	291 (41.38)	53 (9.15)	1 (0.12)	–	–	–

*kdr* Knockdown resistance gene, *NC* not calculated

*P < 0.05

^a^CR, Confirmed resistance (mortality < 90%); PR, possible resistance (90% ≤ mortality ≤ 97%); S, susceptible (mortality ≥ 98%)

^b^L1014F represents a mutated allele from leucine to phenylalanine at codon 1014 of the para sodium ion channel gene; L1014C is a mutated allele from leucine to cysteine; L1014W leads to a leucine to tryptophan substitution; and L1014L is the wild-type allele

### Genetic diversity analysis of the COI gene of deltamethrin-resistant and deltamethrin-sensitive mosquitoes

Genetic diversity analysis of 58 COI gene sequences of mosquitoes collected in the 2020–2021 study period showed that there were 59 mutation sites, including 27 singleton variable sites and 32 parsimony informative sites. As shown in Table 5, the total haploid diversity (Hd ± standard deviation) was 0.953 ± 0.022 according to the analysis of the DNA diversity index in 2021, and the haploid diversity index (Hd) ± SD was 1.00 in 2020. The total nucleotide diversity was 0.00910 (in 2021) and 0.00864 (in 2020). The results of Tajima's *D* and of Fu and Li's *D* and* F* were not significantly negative from 2020–2021 (*P* > 0.10). The genetic distance between resistant and sensitive mosquito populations was 0.009, but their intraspecific genetic distances were 0.009 and 0.01, indicating a close genetic relationship between the two populations. The genetic differentiation index (*Fst*) between the two populations was not significantly negative (*P* > 0.10), indicating no significant genetic differentiation between the two populations (Additional file 2: Table S2).

**Table 5:** Genetic diversity analysis of mosquitoes collected in Xiangcheng from 2020 to 2021

Genetic diversity index	Populations		Populations in 2021 (total* n*)	Populations in 2020 (total* n*)
	Deltamethrin-resistant mosquitoes	Deltamethrin-sensitive mosquitoes		
*N*	30	22	52	6
Segregating sites	34	35	46	13
Singleton variable sites	14	19	18	9
Parsimony informative sites	20	16	28	4
Haplotypes	22	18	36	6
Hd ± SD	0.956 ± 0.027	0.957 ± 0.037	0.953 ± 0.022	1.00
Nucleotide diversity	0.00880	0.00964	0.00910	0.00864
Average nucleotide difference	5.88046	6.43723	6.07843	5.2
Tajima's *D*	− 1.22150*	− 1.35174*	− 1.42173*	− 0.52988*
Fu and Li's *D*	− 1.13065*	− 1.58763*	− 1.37607*	− 0.52634*
Fu and Li's *F*	− 1.36796*	− 1.77137*	− 1.66142*	− 0.57146*

*Hd* Haploid diversity index,* SD* standard deviation

*P > 0.1

**Fig. 1:**
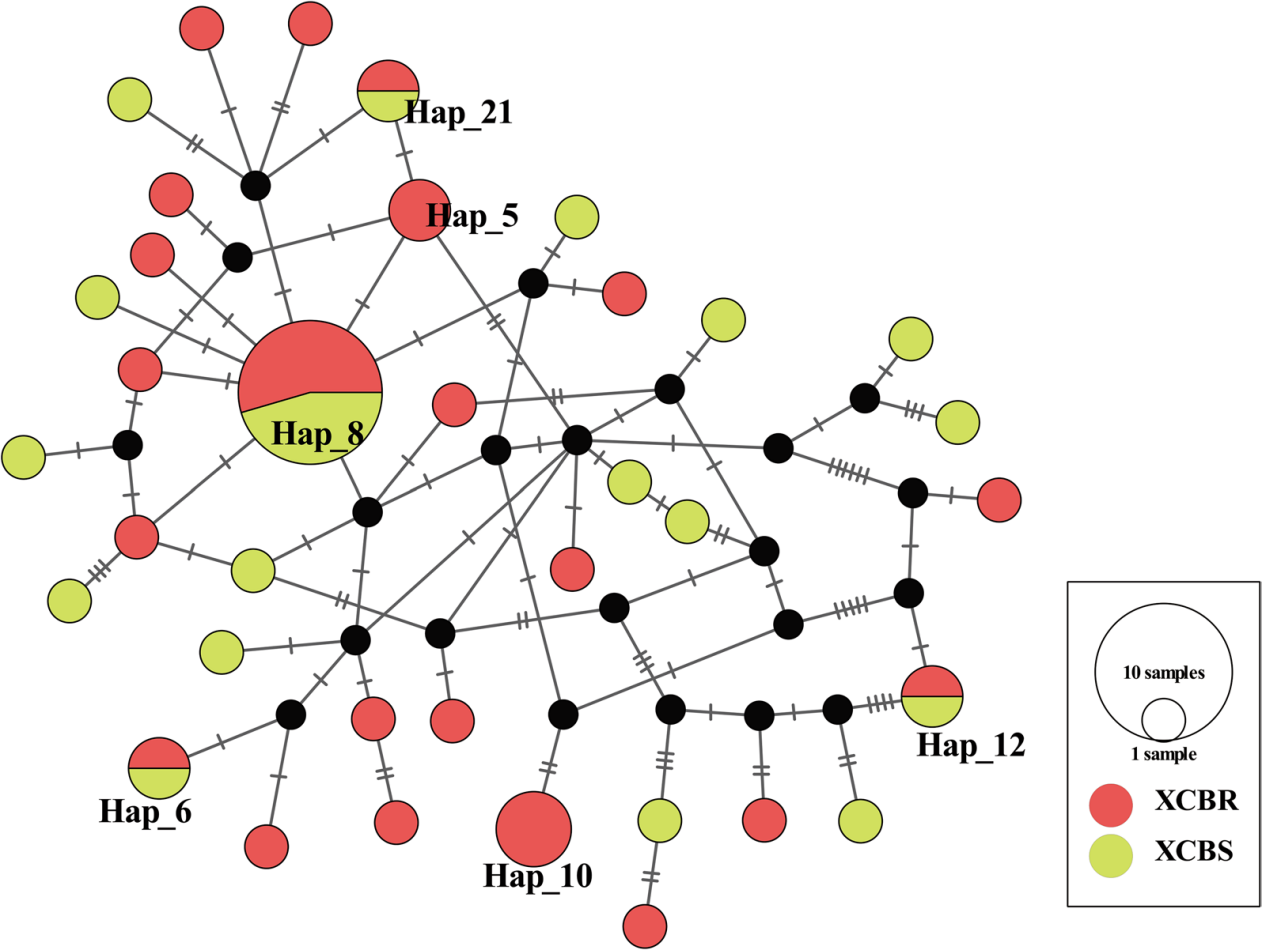
Haplotype network of deltamethrin-resistant and deltamethrin-sensitive mosquitoes. Circles represent a haplotype, black dots represent the assumed intermediate haplotype and the horizontal line represents a mutation step. The size of the circle is proportional to the frequency of the haplotype

A total of 36 haplotypes were detected in 52 mtDNA-COI gene sequences of mosquitoes collected in 2021, including 32 unique haplotypes and four shared haplotypes. Among the 32 unique haplotypes, 18 were from resistant mosquitoes and 14 were from sensitive mosquitoes. The haplotype network diagram shows that shared haplotype Hap_8 accounted for the largest number (6 resistant mosquitoes, 5 sensitive mosquitoes) of mosquitoes, and haplotypes Hap_6, Hap_12 and Hap_ 21 were shared by two populations, but there were only two. As shown in Fig. 1, each haplotype was interlaced and did not form two distinct branches.

## Discussion

Molecular monitoring and bioassays of insecticide resistance status in local malaria vectors are important tools for managing and controlling resistance vectors [30]. A previous study demonstrated the initial development of resistance in *An. sinensis* to deltamethrin and cyfluthrin in Nanyang City [31]. The results of Hu et al.’s study indicate that resistance of *An. sinensis* to deltamethrin and beta-cyfluthrin in Puyang City occurred in 2018 and 2020 [20]. The results of resistance monitoring in our study showed that the resistance of *An. sinensis* to pyrethroid insecticides in Henan Province is increasing, with the level of resistance to the propoxur insecticide already reached in the four studied regions; however, the resistance to malathion was different in terms of sensitivity to resistance. The results for malathion are similar to those reported in previous studies [20, 21], possibly due to the lower use of malathion in Henan Province in recent decades [32]. Given this present-day sensitivity of mosquitoes to malathion, alternative insecticides can be used to prolong the development of mosquito resistance to malathion.

In our study, only one mutation type, G119S, was found. This result is in line with observations reported in published studies suggesting that the G119S mutation is widely distributed in *An. sinensis* [33]*.* Compared to other mosquitoes, this result is also consistent with the *ace* gene mutations in *Anopheles gambiae* and *Culex pipiens* [34]*.* Qin et al. reported a modest to high (45–75%) *ace-1* mutation frequency in *An. sinensis* populations [33]. Chang et al. demonstrated that the frequencies of G119S mutations in the Anhui (China) and Yunnan (China) populations were 58.9% and 38.5%, respectively [35]. These results suggest that the G119S mutation rate of *An. sinensis* is high and that this mutation is widely distributed. A previous study revealed that the G119S allele frequency of a homozygous mutation was 100% in mosquitoes from the two *An. sinensis* populations, indicating that it is not involved in malathion resistance [36]. The gene mutation rate of the Xiangfu population in Henan Province was as high as 54.74%, indicating that the high frequency of the G119S mutation might be the result of the long-term use of organophosphorus insecticides for agricultural pest control [27]. The high frequency of the G119S mutation indicated that these *An. sinensis* populations have a high risk of developing resistance to organophosphorus and carbamate insecticides. Other researchers found that the G285A and F350Y mutations on the *ace* gene are related to resistance to organic phosphorus, and that the F105S, G285A and F305Y mutations are related to resistance to carbamate [37]. Higher G119S allele frequencies were observed in both propoxur- and malathion-resistant mosquitoes than in propoxur- and malathion-sensitive mosquitoes in the Tanghe population, but such a development was not observed in the other three sites, suggesting that the production of resistance might be the result of the joint action of multiple factors.

*Kdr* was first identified in the house-fly *Musca domestica *L. [38]. The L1014F substitution has been reported in pyrethroid-resistant pest species, including *An. gambiae* and *Cx. p. pallens* [39, 40]. The L1014C mutation, with TTG (Leu) being replaced with TGT (Cys), is a newly reported mutation [41]. The L1014F and L1014C mutations were detected in the Republic of Korea, where the TTC L1014F mutation was observed for the first time [42]. Previous studies revealed that the frequencies of the L1014F allele were significantly associated with deltamethrin-resistant and DDT-resistant phenotypes, but that this was not the case for the L1014C allele. In Kaifeng (Henan, China), the difference in *kdr* mutation frequency between surviving and dead mosquitoes was statistically significant [36, 43]. However, Sun et al. reported that the difference in *kdr* mutation frequency between surviving and dead mosquitoes was not statistically significant in Yingjiang (Yunnan, China) and Suining (Jiangsu, China) [43].

 Three types of *kdr* mutant alleles were found in the present study, including L1014F, L1014C and L1014W. In contrast to the situation with *An. sinensis* in Sichuan, China, where the frequency of L1014F was observed to be higher than that of L1014L. L1014L was found to be the predominant resistance allele in Henan, but L1014C had lower frequencies than that L1014F and L1014L, which is similar to the results of the present study [33]. L1014F and L1014C mutations were found in Henan Province in a previous study, and the L1014W mutation was first found in the present study [11]. In the present study, the proportion of the L1014W mutation was very small (0.12%) and only distributed in the Pingqiao population. Tan et al. reported that the presence of the L1014W mutation in 52 *An. sinensis* in Guangxi, based on their *kdr* gene mutation study, accounting for a small proportion (1%) [44]. This result is similar to that of the current study. Higher L1014F and lower L1014C allele frequencies were observed in beta-cyfluthrin-resistant mosquitoes than in beta-cyfluthrin-sensitive mosquitoes, suggesting that the relationship between higher L1014F and lower L1014C allele frequencies and insecticide resistance requires further in-depth analysis.

The evolution of insect resistance is based on the genetics of insecticide-resistant populations. The Hd was > 0.95, and nucleotide diversity was > 0.008 from 2020–2021, indicating that the overall genetic diversity of *An. sinensis* in Henan Province is high. The values of Tajima's *D* and of Fu and Li's *D* and *F* suggest that the populations conform to the neutral selection hypothesis and are not subject to obvious selection pressure during the evolution process. Yang et al. studied the population genetic variation and population structure characteristics of five species groups of *An. sinensis* in nine sampling sites in Yunnan Province [45]. These authors reported gene exchange between the four species groups of *An. sinensis* in Yunnan Province, except for the YU population group, and that genetic differentiation was not apparent [45]. The values of the *Fst* and the haplotype network diagram indicated that there was no genetic differentiation between deltamethrin-resistant and deltamethrin-sensitive mosquito populations, possibly because the mosquitoes in this study were all adult mosquitoes collected in the field, and they lived in the same ecological environment and faced the same pressure of insecticide selection. External environmental factors might neutralize the degree of genetic differentiation, so genetic differentiation between the two populations was not observed. The results of this study show that the emergence of mosquito resistance might be the result of multiple mechanisms.

The advantages of adult female mosquitoes directly captured in the field for the study of insecticide resistance are that fewer facilities are required, and the age distribution of the vectors is representative of the wild vector population at a given time and location. The limitations of this study include failing to accurately assess adult female mosquitoes’ blood-sucking history, age and survival status, any of which might affect the test results. No further study on mutations at other sites besides the *kdr* and *ace-1* genes and no comprehensive study of resistance mechanisms have been carried out from the perspective of enzymology and proteomics.

## Conclusions

Four *Anopheles* species were identified in the present study: *An. sinensis*, *An. yatsushiroensis*, *An. anthropophagus* and *An. belenrae*. *Anopheles belenrae* was discovered for the first time in Henan Province. A high resistance to pyrethroids and propoxur was noted in the mosquitoes collected in Henan Province, but resistance to malathion varied according to the sampling location. The L1014W (TGG) mutation was detected for the first time in this study. Only one mutation, G119S, was found in the *ace-1* gene. Mutations in the *kdr* and *ace-1* genes play a role in the generation of insecticide resistance in *An. sinensis*. The genetic diversity of deltamethrin-resistant and deltamethrin-sensitive mosquitoes showed no genetic differentiation. The generation of resistance might be the result of the combination of multiple factors. Further experiments are needed to collect comprehensive research data on resistance mechanisms from the perspectives of enzymology and proteomics. These findings are a step towards providing missing data on the insecticide resistance of *An. sinensis* in Henan Province and provide the scientific basis for guiding the control of *An. sinensis*.

## Supplementary Information

**Additional file 1: Table S1**. Distribution and frequency of 10 genotypes at 1014 in the *kdr* gene in *Anopheles sinensis*.

**Additional file 2: Table S2.** Genetic distance and genetic differentiation index of the two populations.

## Data Availability

The datasets used during the current study are available from the corresponding author on reasonable request.

## Abbreviations

*ace-1*: Acetylcholinesterase-1
CDC: Center for Disease Control and Prevention
COI: Cytochrome oxidase subunit I
CR: Confirmed resistance
GABA: Gamma-aminobutyric acid
*kdr*: Knockdown resistance
mtDNA: Mitochondria DNA
PR: Possible resistance
S: Susceptible
SC: Sodium ion channels
