# Supplementary material for: Insecticide resistance of Anopheles sinensis after elimination of malaria in Henan Province, China
Source: Parasit Vectors. 2023 Jun 2;16:180. doi: 10.1186/s13071-023-05796-z (PMC10239179; doi:10.1186/s13071-023-05796-z)
Supplement: Supplementary file 1 — Additional file 1: Table S1. Distribution and frequency of 10 genotypes at 1014 in the kdr gene in Anopheles sinensis. [file 13071_2023_5796_MOESM1_ESM.docx]

**Table S1** Distribution and frequency of 10 genotypes at 1014 in the *kdr* gene in *Anopheles sinensis*

| Genotype | Pingqiao | Tanghe | Xiangcheng | Xiangfu | Frequency (%) |
| --- | --- | --- | --- | --- | --- |
| TTG (L/L) | 13 | 52 | 66 | 0 | 38.64% |
| TTT (F/F) | 28 | 3 | 1 | 57 | 26.25% |
| TGT (C/C) | 1 | 0 | 0 | 1 | 0.59% |
| TTG/TGT (L/C) | 8 | 2 | 11 | 1 | 6.49% |
| TGG/TTT (W/F) | 1 | 0 | 0 | 0 | 0.29% |
| TTT/TGT (F/C) | 11 | 5 | 2 | 7 | 7.37% |
| TGT/TTC (C/F) | 2 | 0 | 0 | 0 | 0.59% |
| TTG/TTT (L/F) | 19 | 7 | 18 | 4 | 14.16% |
| TTG/TTC (L/F) | 0 | 1 | 0 | 0 | 0.29% |
| TTT/TTC (F/F) | 4 | 0 | 0 | 14 | 5.31% |
| Total | 87 | 70 | 98 | 84 | 100.00% |
