# Supplementary material for: Insecticide resistance of Anopheles sinensis after elimination of malaria in Henan Province, China
Source: Parasit Vectors. 2023 Jun 2;16:180. doi: 10.1186/s13071-023-05796-z (PMC10239179; doi:10.1186/s13071-023-05796-z)
Supplement: Supplementary file 2 — Additional file 2: Table S2. Genetic distance and genetic differentiation index of the two populations. [file 13071_2023_5796_MOESM2_ESM.docx]

**Table S2** Genetic distance and genetic differentiation index of the two populations

| Populations | Deltamethrin resistant mosquitoes | Deltamethrin sensitive mosquitoes | *P* value |
| --- | --- | --- | --- |
| Deltamethrin resistant mosquitoes | 0.009 | -0.012 | > 0.10 |
| Deltamethrin sensitive mosquitoes | 0.009 | 0.01 |  |

Note：On the diagonal is intraspecific genetic distance, and below the diagonal is interspecific genetic distance.
